# Fresh *Gastrodia elata* Blume alleviates simulated weightlessness-induced cognitive impairment by regulating inflammatory and apoptosis-related pathways

**DOI:** 10.3389/fphar.2023.1173920

**Published:** 2023-05-03

**Authors:** Yiwen Zhang, Hong Huang, Caihong Yao, Xinran Sun, Qinghu He, Muhammad Iqbal Choudharyc, Shanguang Chen, Xinmin Liu, Ning Jiang

**Affiliations:** ^1^ Research Center for Pharmacology and Toxicology, Institute of Medicinal Plant Development (IMPLAD), Chinese Academy of Medical Sciences and Peking Union Medical College, Beijing, China; ^2^ Sino-Pakistan Center on Traditional Chinese Medicine, Hunan University of Medicine, Huaihua, China; ^3^ H.E.J. Research Institute of Chemistry, International Center for Chemical and Biological Sciences, University of Karachi, Karachi, Pakistan; ^4^ National Laboratory of Human Factors Engineering, The State Key Laboratory of Space Medicine Fundamentals and Application, China Astronaut Research and Training Center, Beijing, China; ^5^ Institute of Drug Discovery Technology, Ningbo University, Ningbo, China; ^6^ Healthy & Intelligent Kitchen Engineering Research Center of Zhejiang Province, Zhejiang, China

**Keywords:** *Gastrodia elata* Blume, stimulated weightlessness, learning and memory, inflammatory, apoptosis

## Abstract

In aerospace medicine, the influence of microgravity on cognition has always been a risk factor threatening astronauts’ health. The traditional medicinal plant and food material *Gastrodia elata* Blume has been used as a therapeutic drug for neurological diseases for a long time due to its unique neuroprotective effect. To study the effect of fresh *Gastrodia elata* Blume (FG) on cognitive impairment caused by microgravity, hindlimb unloading (HU) was used to stimulate weightlessness in mice. The fresh *Gastrodia elata* Blume (0.5 g/kg or 1.0 g/kg) was intragastrically administered daily to mice exposed to HU and behavioral tests were conducted after four weeks to detect the cognitive status of animals. The behavioral tests results showed that fresh *Gastrodia elata* Blume therapy significantly improved the performance of mice in the object location recognition test, Step-Down test, and Morris Water Maze test, including short-term and long-term spatial memory. According to the biochemical test results, fresh *Gastrodia elata* Blume administration not only reduced serum factor levels of oxidative stress but also maintained the balance of pro-inflammatory and anti-inflammatory factors in the hippocampus, reversing the abnormal increase of NLRP3 and NF-κB. The apoptosis-related proteins were downregulated which may be related to the activation of the PI3K/AKT/mTOR pathway by fresh *Gastrodia elata* Blume therapy, and the abnormal changes of synapse-related protein and glutamate neurotransmitter were corrected. These results identify the improvement effect of fresh *Gastrodia elata* Blume as a new application form of *Gastrodia elata* Blume on cognitive impairment caused by simulated weightlessness and advance our understanding of the mechanism of fresh *Gastrodia elata* Blume on the neuroprotective effect.

## Introduction

Astronauts’ cognitive impairment caused by weightlessness has always been an urgent problem to be solved. Evidence has proved that after experiencing the microgravity and confined environments of spaceflight, astronauts are at an increased risk of memory impairment, disorientation, and other symptoms (Casler and Cook, 1999). The pressure of long-term space flight would lead to the cognitive overload of astronauts, causing damage to the completion of the mission (Bock et al., 2010; Lv et al., 2021). Especially, evidence showed that the operational tasks in space missions are more challenging for humans compared with ground missions (Fowler et al., 2000). In space experiments of animals, it was found that long-term spaceflight affects the principal regulatory factors of brain neuroplasticity and neurotrophic factors in rodents (Popova et al., 2020). Combined with these conditions, maintaining the health of astronauts in long-term space flight has become one of the main concerns of aerospace medicine. This decline in learning and memory abilities caused by specific circumstances is a functional impairment, with no clear lesion location and specific targets and no effective prevention and treatment methods. Although in 1990, NASA launched the “Neurolab Mission” hoping to find measures to protect astronauts from cognitive decline caused by aerospace stress, the progress has been slow so far (Homick et al., 1998). Finding safe and effective protective measures to improve and increase the response and decision-making abilities of astronauts in special aerospace environments remain a challenge facing the international aerospace medical community. To better study the effects of microgravity on the human body, hindlimb unloading caused by tail suspension is used as a classic modeling method to simulate weightlessness in the present pharmacological research, which has been proven to take risk of learning and memory impairment in rodents (Globus and Morey-Holton, 2016).

Traditional Chinese Medicine (TCM) places more emphasis on the holistic concept of diseases, and the concept of “treating diseases before they occur” in TCM is more suitable for preventing functional injury. *Gastrodia elata* Blume, both as a traditional medicinal plant and food material, is often used to treat dizziness, headache, and cognitive impairment caused by Alzheimer’s disease, ischemic brain injury, etc. (Mao et al., 2017; Fasina et al., 2022). *In vivo* and *vitro* experiments, *Gastrodia elata* Blume shows protective effects on neuronal cells from oxidative stress, apoptosis, and inflammatory responses, thus, improving cognitive dysfunction induced by various brain injury models (Ng et al., 2016; Zhou et al., 2018; Lin et al., 2021). In this research, water extract of Fresh *Gastrodia elata* Blume (FG) was used, which is a new and original form that maintains its original nutritional value compared with the processed form and is commonly used in herbal cuisine (Huang et al., 2021). Moreover, our previous research has proved that FG has great benefits for improving cognitive impairment caused by circadian rhythm disorder and chronic restraint stress in mice, both of which are problems faced by astronauts during spaceflight (Huang et al., 2021; Huang et al., 2022). The present study was designed to investigate the effect of FG on cognitive impairment induced by a simulated weightlessness model to explore the potential application value of FG in aerospace medicine.

## Materials and methods

### Animals

Male ICR mice, weighing 23–25 g, were obtained from Charles River Laboratories, Beijing, China, with Qualified No. SCXK 2012-0001. All mice were housed, with a maximum of five mice per cage and a 12:12 h light/dark cycle (lights on at 8:00 a.m.). The room temperature and humidity condition were maintained at 23°C ± 2°C and 55% ± 10%. Before experiments, mice were adapted to the environment for 7 days, and all the behavioral experiments were conducted during the light phase. The animals were divided into two batches for testing different behavioral experiments, respectively, with twelve animals in each group. The protocol described in the present study was approved by the committee for the Care and Use of Laboratory Animals of the Institute of Medicinal Plant Development, Beijing, China, (NO. 20161028).

### Drugs

The fresh *Gastrodia elata* Blume tuber used in this experiment was purchased from its authentic origin—Jinkouhe, Sichuan Province, China, and identified as the tuber of *Gastrodia elata* Blume by Guanghua Lu, professor at Chengdu University of Traditional Chinese Medicine, Chengdu, China. The manufacturing process of the FG sample is consistent with previous literature and the content of GAS and HBA in FG has been determined with the HPLC chromatogram of the reference material published (Huang et al., 2021; Huang et al., 2022). After being crushed by the high-speed blender, we collected the filtrate and washed the filter residue with purified water to ensure complete extraction. The collected liquid was freeze-dried and refrigerated at −20°C for later use. Donepezil hydrochloride (DNP [Aricept], Eisai Inc. [Ibaraki, Japan]) was used as a positive control.

### Treatment

**FIGURE 1:**
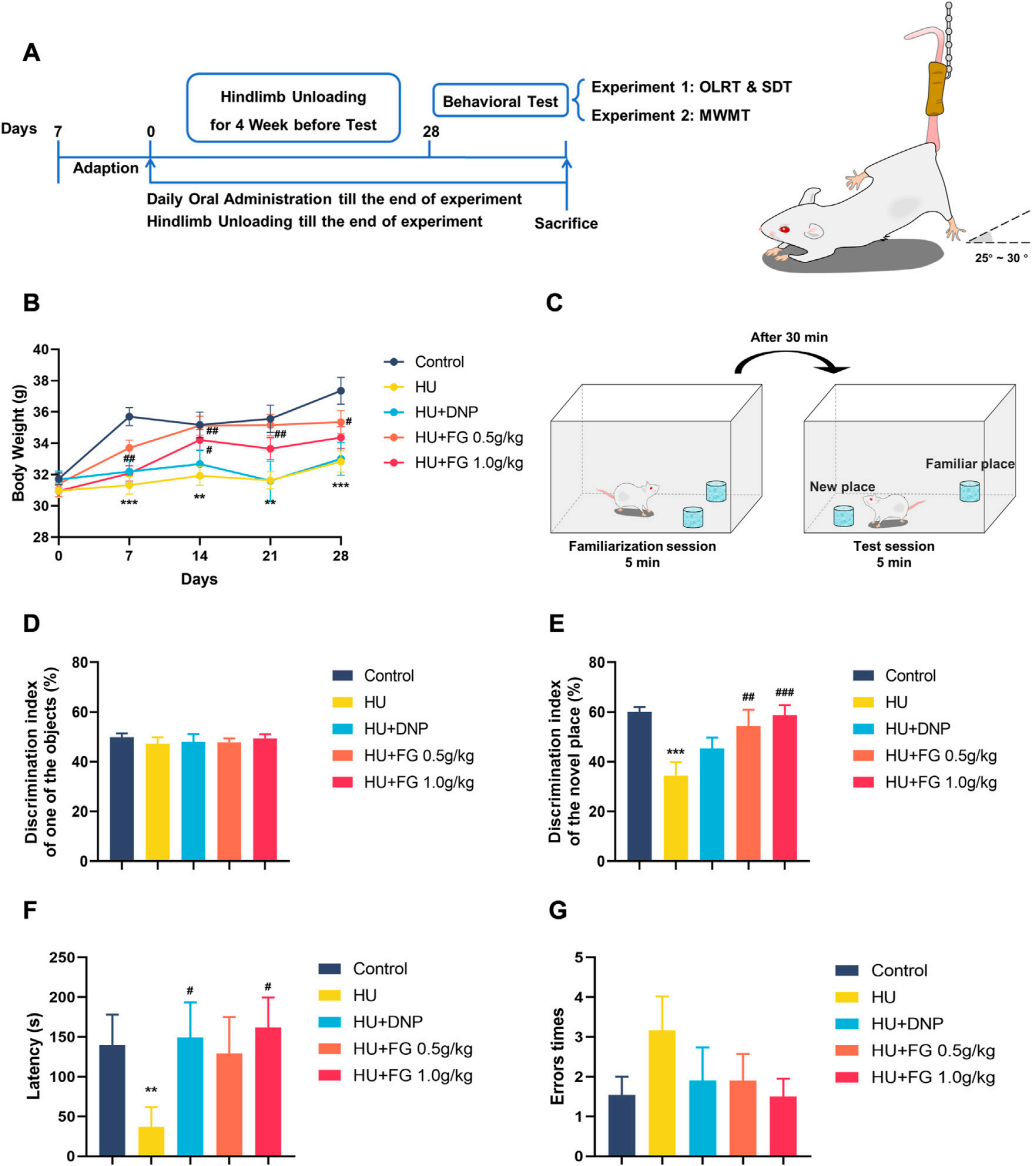
The schematic diagram of the experiment and the effect of FG on LORT and STD. **(A)** The experimental protocol of this study. **(B)** Body weight change during the HU procedure. **(C)** Schematic diagram of OLRT. **(D)** The discrimination index in the familiarization session of LORT. **(E)** The discrimination index in the test session of LORT. **(F)** The latency SDT. **(G)** The error times of SDT. Data were expressed as mean ± SEM (n = 10–12 per group). *p < 0.05, **p < 0.01, and ***p < 0.001 versus the control group; ^#^ *p <* 0.05, ^##^ *p <* 0.01, and ^###^ *p <* 0.001 versus the HU group. Note: Hindlimb unloading, HU; Object location recognition test, OLRT; Step Down test, SDT; Morris Water Maze Test, WMWT.

Animals were randomly divided into five groups, namely, the control group, model group, positive drug group (Donepezil, 1.6 mg/kg), FG low-dose group (0.5 g/kg), and high-dose group (1.0 g/kg). The dose of Donepezil was determined by previous literature (Yang et al., 2020), and the doses of FG were according to our preliminary experiments and literature reports (Huang et al., 2021; Huang et al., 2022). Oral administration was at a volume of 20 mL/kg and started at the same time as the modeling. Drugs were formulated into corresponding concentration liquids with distilled water according to the above-mentioned dosages. The control group and model group were given corresponding volumes of distilled water. Modeling and drug administration continued until the end of behavioral testing (Figure 1A).

### Hindlimb unloading procedures (HU)

A simulated weightlessness apparatus was developed to keep the hindlimb of mice off the ground with the body at a 25°–30° angle for a long time, allowing free access to water and food (Chinese patent No. 201310228949.2). Briefly, mice were placed in a 26 cm × 26 cm × 30 cm black plexiglass box with their tails bound by medical adhesive tape and hung with a small hook in a stainless chain mounted at the top of the cage. Mice were isolated from each other and allowed free access to water and food. The animals remained tail-suspended for 28-day modeling except for daily drug administration.

### Behavioral tests

#### Object location recognition test, OLRT

The animals were allowed to explore the experimental test box (45 L × 45 W × 30 H cm) once a day (10 min for each session) for a 3-day adaptation period before familiarization period and testing period were operated. Two identical objects were put in a symmetrical position on one side of the chamber, allowing animals to explore for 5 min. After a 30-min intersession interval, one of the familiar objects was moved to the contralateral position. To avoid biasing the experimental results due to the animal’s position preference, the familiar position and the new position were in balance during the test period. The memory ability of animals is evaluated by the discrimination index (DI). The calculation formula is DI = (T_N_)/(T_N_ + T_F_) × 100%. T_N_ (new) and T_F_ (familiar) means the exploration time of an object in the new position and the exploration time of an object in the familiar position objects, respectively.

#### Step-down Test

Step-Down Test is a memory evaluation method based on the punishment principle. On the first day of the acquisition experiment, the animals acclimated for 3 min in the chamber (20 L × 12 W cm), and then the electric grid at the bottom of the chamber gave a continuous current of 0.3 mA for 5 min. Animals can escape the shock by jumping onto an insulated platform located on one side of the chamber. A retention test was conducted after 24 h lasting 5 min. Animals were placed on the insulated platform, and the power grid was immediately energized. The latency and total error times of the animals jumping off the platform were recorded.

#### Morris water maze test

The experiment is divided into three stages: the positioning navigation training session, the space exploration session, and the working memory experiment. In the 5-day positioning navigation training, the animals were put into the pool (120 D × 40 H cm) from different quadrants twice a day and the fixed platform was hidden 1.5 cm below the water surface. The space exploration test was carried out on the sixth day, in which the platform was removed. In the working memory experiment, the platform was placed and moved into adjacent quadrants sequentially and carried out for 3 days to test the working memory of animals.

### Biochemical Analysis

#### Preparation of serum and brain samples

All mice were sacrificed the day after the last behavioral tests to collect the biological samples. Blood was collected from the ophthalmic veins and stood at 4°C overnight to obtain the serum. Three mice were randomly selected and transcardially perfused for Nissl staining. The hippocampi were dissected on ice and stored at −80°C until analysis.

#### Determination of biochemical parameters

The levels of SOD, MDA, and GSSG in the serum were detected using commercial kits from Beyotime (Shanghai, China) according to the manufacturer’s protocols. The levels of TNF-*α*, IFN-*γ*, IL-4, IL-6, IL-10, and Arg-1 of the hippocampal were determined by commercial enzyme-linked immunosorbent assay (ELISA) kits from Dakewe Biotech (Shenzhen, China) according to the manufacturer’s protocols.

#### Western blotting analysis

The hippocampus was homogenized in protein lysis buffer (Solarbio, China) and fully lysed for 30 min. After centrifuging (12 000 g, 4°C, and 30 min), the supernatant was taken. The protein concentration was detected by BCA protein assay kits (CWBIO, China) and was prepared to 5 μg/μL protein solution with the lysate solution and SDS PAGE loading buffer (×5) for use. Proteins were separated by SDS PAGE, transferred onto PVDF membrane (Merck Millipore, Germany), and then blocked with 5% non-fat milk with Tris Buffered Saline Tween (TBST) for 1.5 h at room temperature. The membranes were incubated with primary antibodies: NLRP3 (1:1,000, Abcam, United Kingdom, #ab263899), NF-κB p65 (1:1,000, Abcam, United Kingdom, #ab19870), BAX (1:1,000, Abcam, United Kingdom, #ab32503), Cyt C (1:1,000, Abcam, United Kingdom, #ab133504), Drp 1, (1:1,000, Abcam, United Kingdom, #ab184247), PI3K (1:1,000, ABclonal, CN, #A19742), AKT (1:1,000, Cell Signaling, United States, #4685); mTOR (1:1,000, Abcam, United Kingdom, #ab32028), SYP (1:1,000, Abcam, United Kingdom, #ab32127), TrkB (1:1,000, Abcam, United Kingdom, #ab187041), and GAPDH (1:1,000, ABclonal, CN, #A19056) at 4°C overnight followed by incubation with HRP-conjugated secondary antibody for 1.5 h at room temperature. The protein bands were visualized by the BeyoECL Moon kit (Beyotime, China). The gray values of band density were analyzed using the Image J software.

#### Nissl’s staining

Three paraffin sections in each group were dewaxed in xylene and rehydrated with graded ethanol (70, 95, and 100%), followed by rehydration with distilled water. Staining was performed according to the Nissl staining kit (Jiancheng Biology, China). The images were obtained using a microscope slide scanner (Pannoramic 250, 3D Histech Ltd., Hungary) and the quantification of integrated optical density (IOD) of Nissl bodies in each group was analyzed with the NIH Image J Pro software (Media Cybernetics, United States).

#### Neurotransmitter detection

The neurotransmitter analysis method was performed as previously described but with minor modifications (Wang et al., 2019). A measurement of 2 μL of the prepared sample was taken for LC-MS/MS analysis. Glu and GABA in the hippocampus were detected by prominence ultrafast liquid chromatography (UFLC) (Shimadzu, Kyoto, Japan) coupled with a QTRAP 5500 mass spectrometer (AB SCIEX, Framingham, MA, United States). The metabolites were separated using the Restek Ultra Aqueous C18 column (100 mm × 2.1 mm, 3 μm, Bellefonte, PA, United States). Gradient elution was performed using 0.1% formic acid and acetonitrile as flow at a rate of 0.4 mL per minute.

#### Statistical analysis

The experimental results were analyzed by the SPSS 21.0 software and performed by the ImageJ and GraphPad Prism Software 5.0. Differences among normally distributed values were analyzed by one-way ANOVA, and LSD was used for the post-test. The Mann-Whitney *U* test was performed to investigate whether the data unfollowed a normal distribution. Data were expressed by mean ± SEM, and it was considered to have a significant difference when *p <* 0.05.

## Result

### FG ameliorated the HS-induced weight loss in mice

The animals were randomly divided into groups with similar weights at the beginning of the experiment and the animals were weighed weekly (Figure 1B). During the hindlimb suspension paradigm, the model group displayed a prominent weight loss (F (4,55) = 8.987, *p <* 0.001). Both the low and high doses (0.5 g/kg and 1.0 g/kg) of FG groups showed significant weight increase in the second week of HU modeling compared with the model group, while DNP administration did not show weight gain effect (F (4,55) = 4.113, *p <* 0.05, and *p <* 0.05 in FG low and high dose group, respectively, *p <* 0.05 in DNP group).

### FG improved the HU-induced position discrimination impairment in the object location recognition test

In the familiarization session, mice showed no preference for any object, while in the test session, the model group showed a significant decrease in the discrimination index compared with the control group (Figures 1C–E) (F (4,50) = 5.615, *p <* 0.001), indicating that the HU modeling-induced mice spent less time exploring the object in the new position than in the old position. DNP treatment increased the relative discrimination index with no significance (*p <* 0.05). A high dose of FG treatment (1.0 g/kg) showed significant improvement in ameliorating the impaired memory ability (*p <* 0.01, *p <* 0.001).

### FG improved the HU-induced memory impairment in the step-down test

In the consolidation stage, compared with the control group, the latency of the HU group was significantly shortened (*p <* 0.01). The DNP group and the high dose (1.0 g/kg) of the FG group showed a significant trend of prolonging the error latency (*p <* 0.05, *p <* 0.05). In addition, compared with the control group, the times of errors in the model group tended to increase, and the number of errors in the administration group was less than that in the model group (Figures 1F, G).

### FG improved the HU-induced spatial and working learning memory impairment in the Morris Water Maze test

**FIGURE 2:**
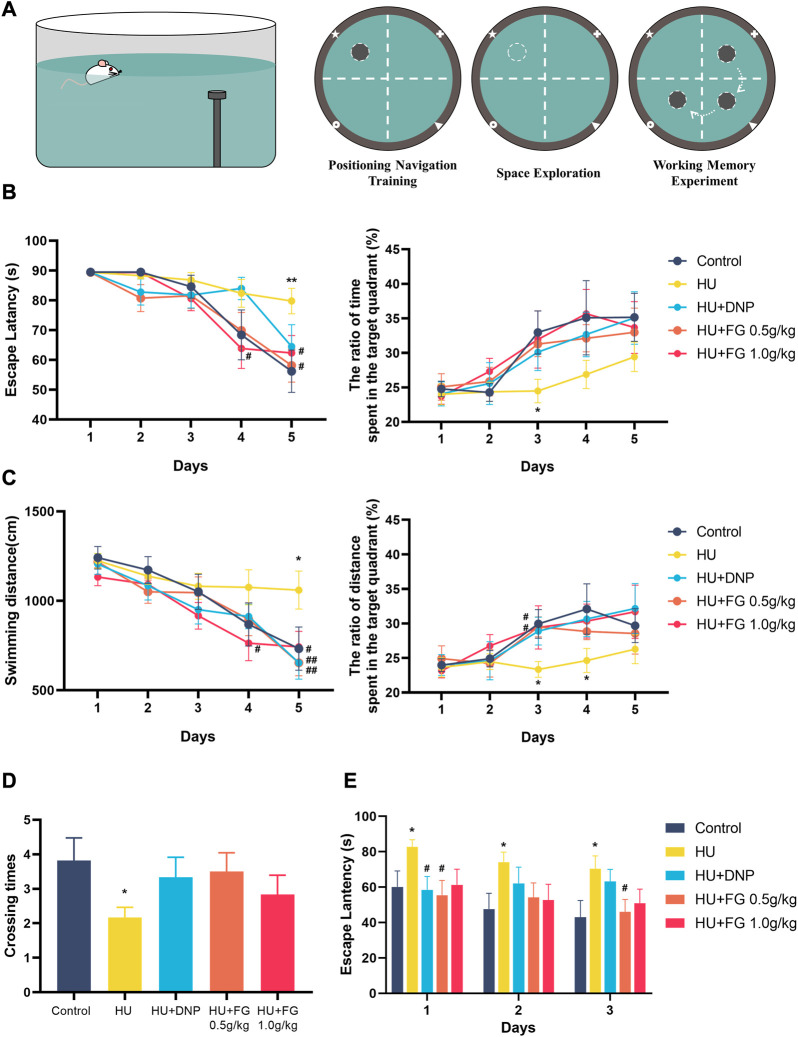
The effect of FG on Morris Water Maze test. **(A)** Schematic diagrams of MWMT. **(B)** The escape latency and the ratio of time spent in the target quadrant during the positioning navigation training session. **(C)** The swimming distance and the ratio of distance spent in the target quadrant during the positioning navigation training session. **(D)** The crossing times in the space exploration session. **(E)** The escape latency in the working memory experiment. Data were expressed as mean ± SEM (n = 10–12 per group). *p < 0.05 and **p < 0.01 versus the control group; ^#^ *p <* 0.05 and ^##^ *p <* 0.01 versus the HU group.

In the positioning navigation training, the HU group had a longer latency and swimming distance for seeking the platform, and there was a significant difference on the fifth day compared with the control group (F (4,54) = 2.332, *p <* 0.01; F(4,54) = 3.118, *p <* 0.05). On the last day of training, both low and high doses (0.5 g/kg and 1.0 g/kg) of FG treatment shortened escape latency (*p <* 0.05 and *p <* 0.05) and swimming distance (*p <* 0.05 and *p <* 0.05). The DNP group also showed a reverse effect but only significantly shortened swimming distance (*p <* 0.05). In addition, the ratio of time and swim distance spent in the target quadrant (where the platform is located) in the HU group was significantly lower than that in the control group (F (4,54) = 1.397, *p <* 0.05, and F (4,54) = 1.770 of the ratio of time and distance, respectively, on the third day; F (4,53) = 1.385 of the ratio of the distance on the fourth day). This impairment was ameliorated by DNP and FG treatment, and on the third day, both low and high doses (0.5 g/kg and 1.0 g/kg) of FG groups showed a significant improvement effect (*p <* 0.05, *p <* 0.05). In the stage of the space exploration experiment, the number of crossing times in the model group was significantly lower than that in the control group (F (4,54) = 1.428, *p <* 0.05). The crossing times in the DNP group and the low and high-dose FG groups showed a higher trend than that in the HU group. During the working memory experiment, the escape latency of the HU group was significantly longer than that in the control group (F (4,52) = 2.117, *p <* 0.05 on the first day; F (4,52) = 1.576, *p <* 0.05 on the second day; and F (4,53) = 2.278 on the last day). The latency of the DNP group decreased significantly on the first day (*p <* 0.05) and the latency of the FG low dose 0.5 g/kg) group decreased significantly on both the first and last days (*p <* 0.05, *p <* 0.05). The others showed a shorter escape latency with no significance (Figure 2).

### FG alleviated oxidative stress in the serum induced by HU

**FIGURE 3:**
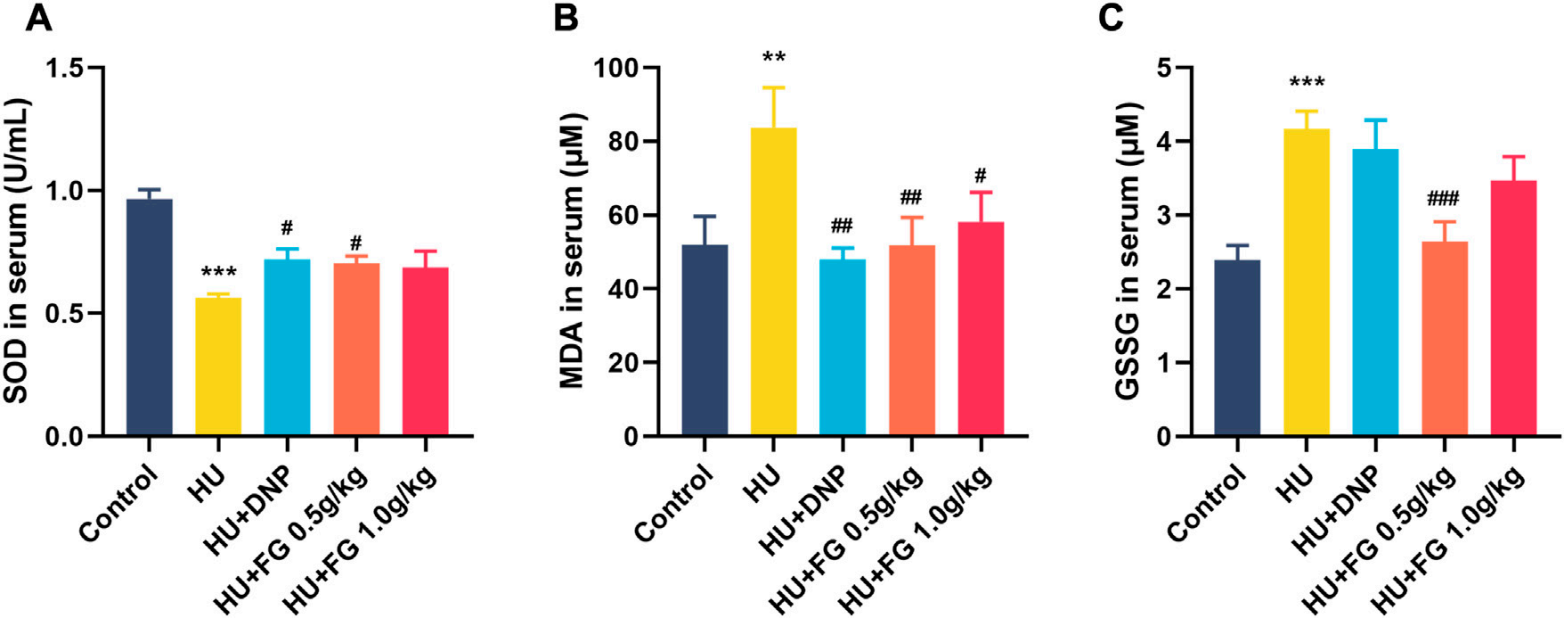
The effect of FG on oxidative stress in the serum. **(A)** The level of SOD. **(B)** The level of MDA. **(C)** The level of GSSG. Data were expressed as mean ± SEM (n = 8–9 per group). **p < 0.01 and ***p < 0.001 versus the control group; ^#^ *p <* 0.05, ^##^ *p <* 0.01, and ^###^ *p <* 0.001 versus the HU group.

In the serum (Figure 3), the production of SOD decreased (F (4,37) = 11.432, *p <* 0.001) and MDA and GSSG increased (F (4,36) = 3.665, *p <* 0.01 in MDA; F(4,35) = 7.312, *p <* 0.001 in GSSG) in HU group. The administration of low-dose FG could significantly reverse the abnormal changes (*p <* 0.05 in SOD, *p <* 0.01 in MDA, and *p <* 0.001 in GSSG). DNP and the high dose FG could also ameliorate the oxidative stress with significance (*p <* 0.05 in SOD and *p <* 0.01 in SOD of the DNP group and *p <* 0.05 in the MDA of the high dose FG group).

### FG reduced inflammatory response in the hippocampus and reversed the upregulation of NF-κB/NLRP3 pathways induced by HU

**FIGURE 4:**
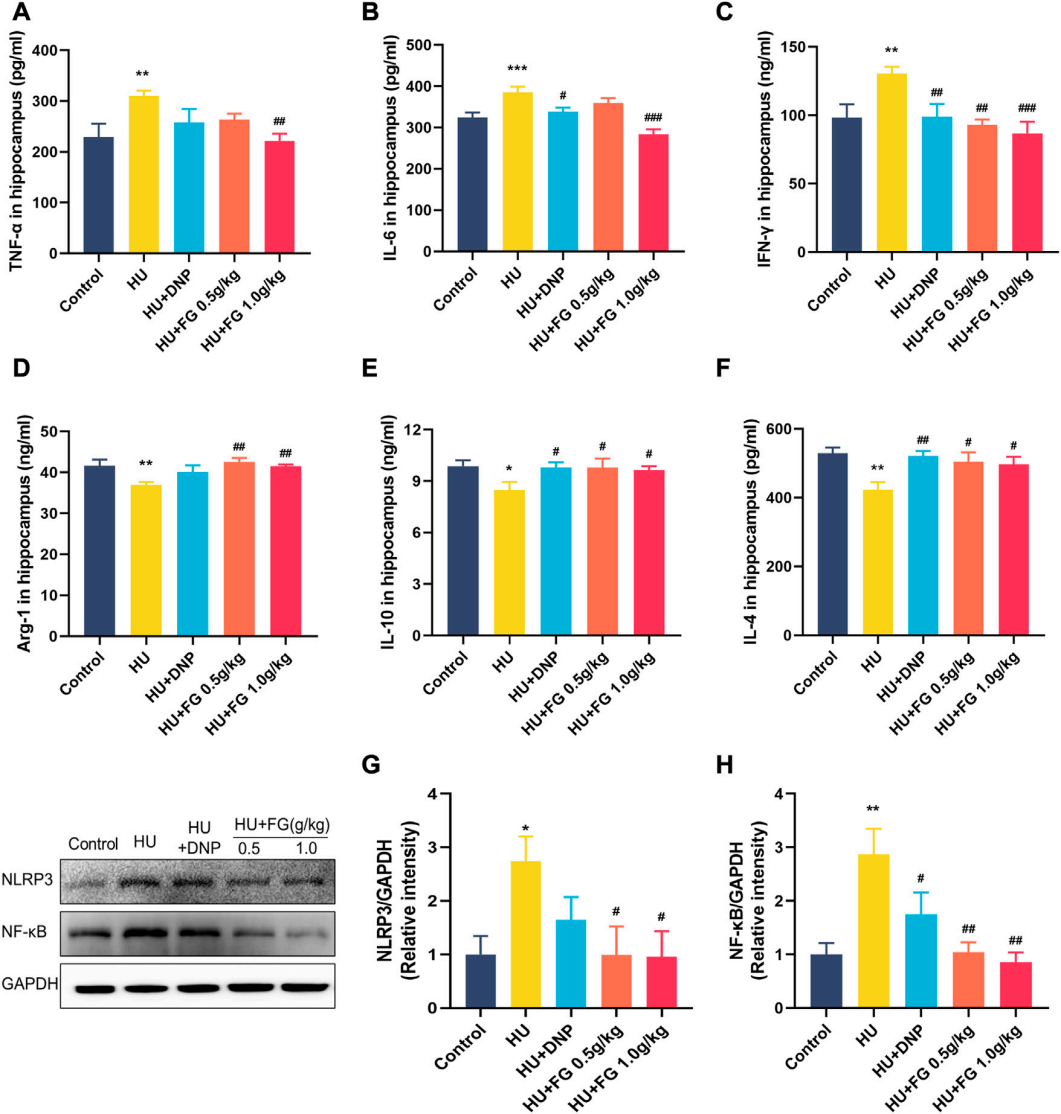
The effect of FG on inflammatory response in the hippocampus. **(A–F)** The levels of TNF-α, IL-6, INF-γ, Arg-1, IL-10, and IL-4 (n = 8–9). **(G, H)** The protein expression of NLRP3 and NF-κB (n = 3). Data were expressed as mean ± SEM. *p < 0.05 and **p < 0.01 versus the control group; ^#^ *p <* .05, ^##^ *p <* 0.01, and ^###^ *p <* 0.001 versus the HU group.

The expressions of three pro-inflammatory factors including TNF-α, IL-6, and IFN-γ were significantly increased in the HU group compared with the control group (F (4,36) = 3.184, *p <* 0.01; F(4,36) = 10.288, *p <* 0.001; F (4,37) = 4.825, *p <* 0.01). The DNP administration significantly reversed the increase of IL-6 and IFN-γ (*p <* 0.05; *p <* 0.01). FG high-dose group showed a significant decrease of TNF-*α*, IL-6, and IFN-*γ* (*p <* 0.01; *p <* 0.001; *p <* 0.01), while the low-dose FG group could decrease them as well but only showed a significant difference in the level of IFN-γ (*p <* 0.01) (Figures 4A-C).

To better explore the effect of FG on the inflammatory response, we measured the levels of anti-inflammatory factors in the hippocampus (Figures 4D-F). The results showed that the levels of Arg-1, IL-10, and IL-4 of the model group were significantly reduced compared with the control group (F (4,37) = 3.707, *p <* 0.01; F(4,37) = 2.250, *p <* 0.05; F (4,35) = 3.898, *p <* 0.01). The administration of DNP significantly increased the levels of IL-10 and IL-4 in the hippocampus (*p <* 0.05; *p <* 0.01), and both low and high doses of FG administration could significantly increase the levels of Arg-1, IL-10, and IL-4 in the hippocampus of mice (*p <* 0.01, *p <* 0.05, and *p <* 0.05 of FG at low dose; *p <* 0.01, *p <* 0.05, and *p <* 0.05 of FG at high dose).

Moreover, to determine the causes of inflammation, the protein expressions of NLRP3 and NF-κB in the hippocampus were measured (Figures 4G-H). The levels of NF-κB and NLRP3 in the hippocampus increased significantly after HU (*p <* 0.05 and *p <* 0.01) and both could be decreased by DNP (*p <* 0.05 in NF-κB) and FG administration (*p <* 0.05 in NLRP3 and *p <* 0.01 in NF-κB).

### FG prevents apoptosis in the hippocampus induced by HU and upregulates the PI3K/AKT signaling pathway

**FIGURE 5:**
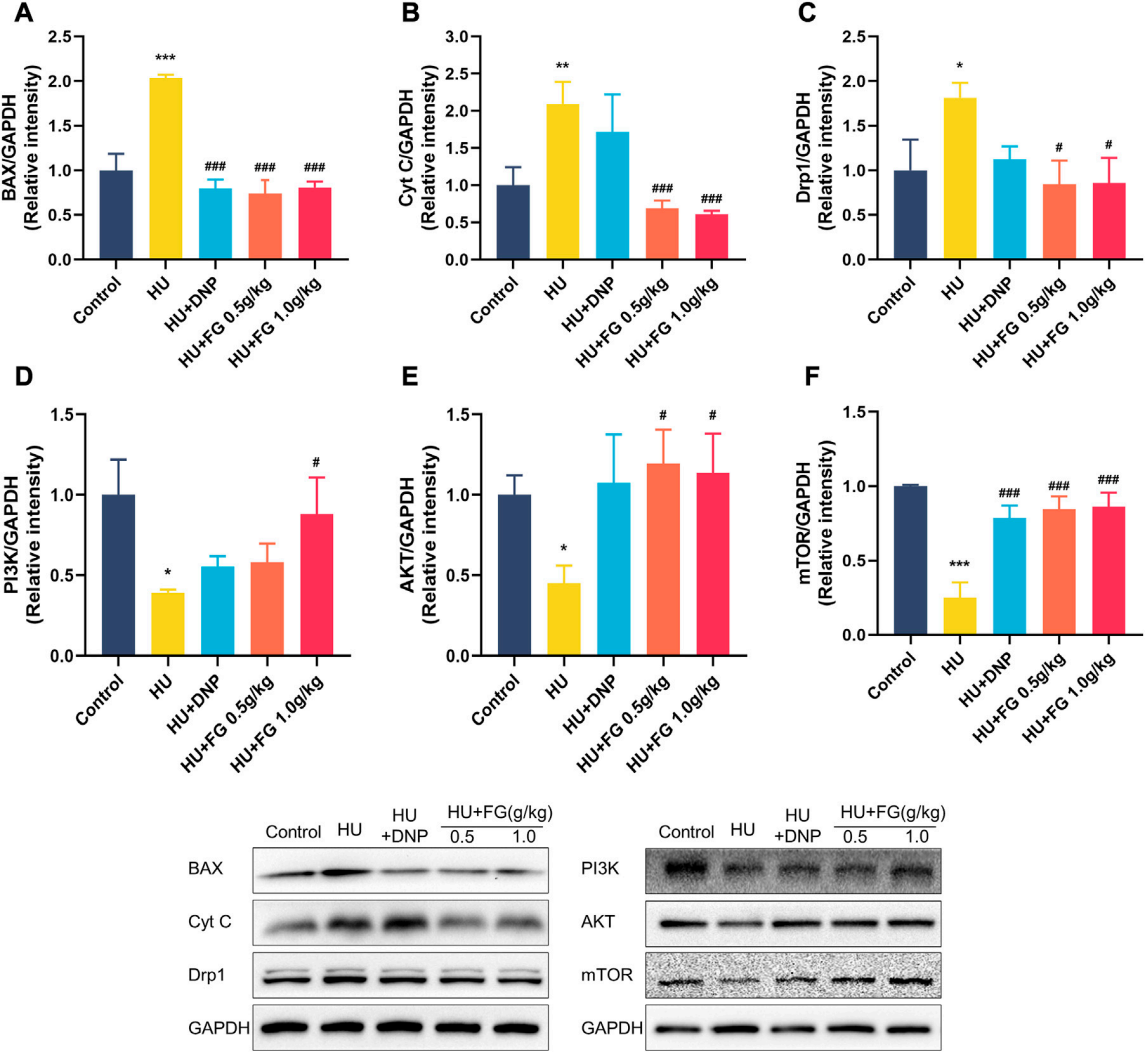
The effect of FG on the anti-apoptotic in the hippocampus. **(A–F)** The protein expression of BAX, Cyt C, Drp1 AKT, PI3K, and mTOR (n = 3). Data were expressed as mean ± SEM. *p < 0.05, **p < 0.01, and ***p < 0.001 versus the control group; ^#^ *p <* 0.05, ^##^ *p <* 0.01, and ^###^ *p <* 0.001 versus the HU group.

In the HU group, the levels of BAX, Cyt C, and Drp1 in the hippocampus were significantly increased (Figures 5A–C; *p <* 0.001, *p <* 0.01, and *p <* 0.05), while treatment with DNP decreased the level of BAX (*p* < 0.001) and FG (0.5 g/kg and 1.0 g/kg) substantially decressed these apoptosis-related proteins (*p <* 0.001, *p <* 0.001, and *p <* 0.05). Figures 5D–F showed the reduced levels of AKT, PI3K, and mTOR in the HU group (*p <* 0.05, *p <* 0.05, and *p <* 0.001), and this reduction could be reversed to varying degrees by the administration of DNP (*p <* 0.001 in mTOR), FG low dose (*p <* 0.05 in AKT; *p <* 0.001 in mTOR), and FG high dose (*p <* 0.05 in PI3K, *p <* 0.05 in AKT, and *p <* 0.001 in mTOR).

### FG improved the hippocampus neuron loss induced by HU

**FIGURE 6:**
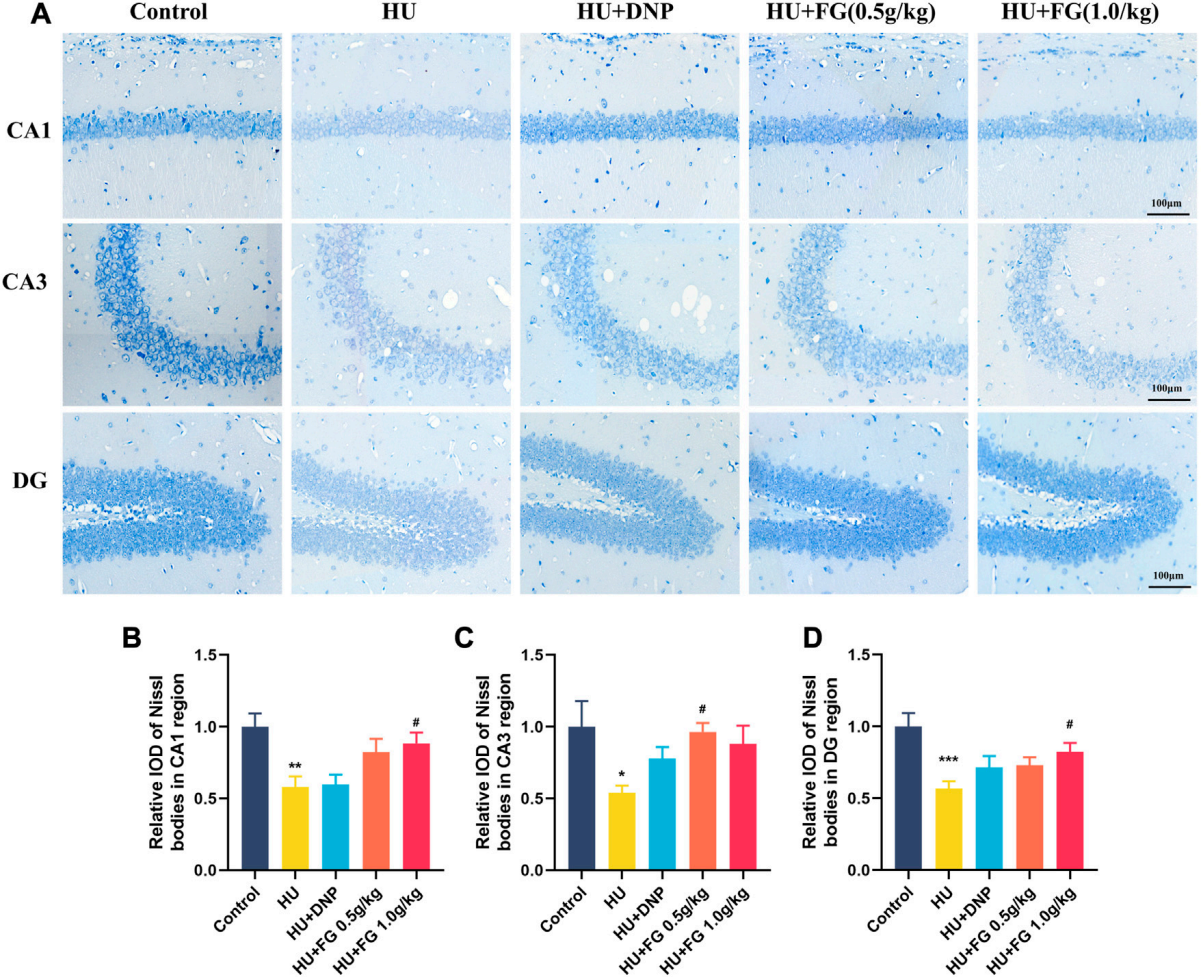
The effect of GRe on the morphological damage to neurons in mice with HU-induced memory impairment. **(A)**The representative Nissl staining photomicrographs of hippocampal CA1, CA3, and DG regions. **(B–D)** The histograms represent the relative IOD values of the Nissl bodies of hippocampal CA1, CA3, and DG regions (n = 3). Data were expressed as mean ± SEM. *p < 0.05, **p < 0.01, and ***p < 0.001 versus the control group; ^#^ *p <* 0.05 versus the HU group.

Since the activation of inflammatory and apoptosis-related factors affects the survival of neurons, neuronal damage was next observed by Nissl’s staining. Figure 6 showed that in the CA1, CA3, and DG subregions of the hippocampus, the Nissl bodies in the control group were clearly stained, and the neurons were abundant and orderly arranged. However, the HS group showed obvious cell loss and loose cell arrangement (*p* < 0.01, *p* < 0.05, and *p* < 0.001 in CA1, CA3, and DG, respectively). While the administration of FG (both low and high doses) could alleviate the neuron loss that occurred in the HU group (*p* < 0.05 and *p* < 0.05 of FG at a high dose in CA1 and DG; *p* < 0.05 of FG at low dose in CA3).

### FG improved synaptic plasticity and maintained the imbalance of GABA/Glu in the hippocampal induced by HU

**FIGURE 7:**
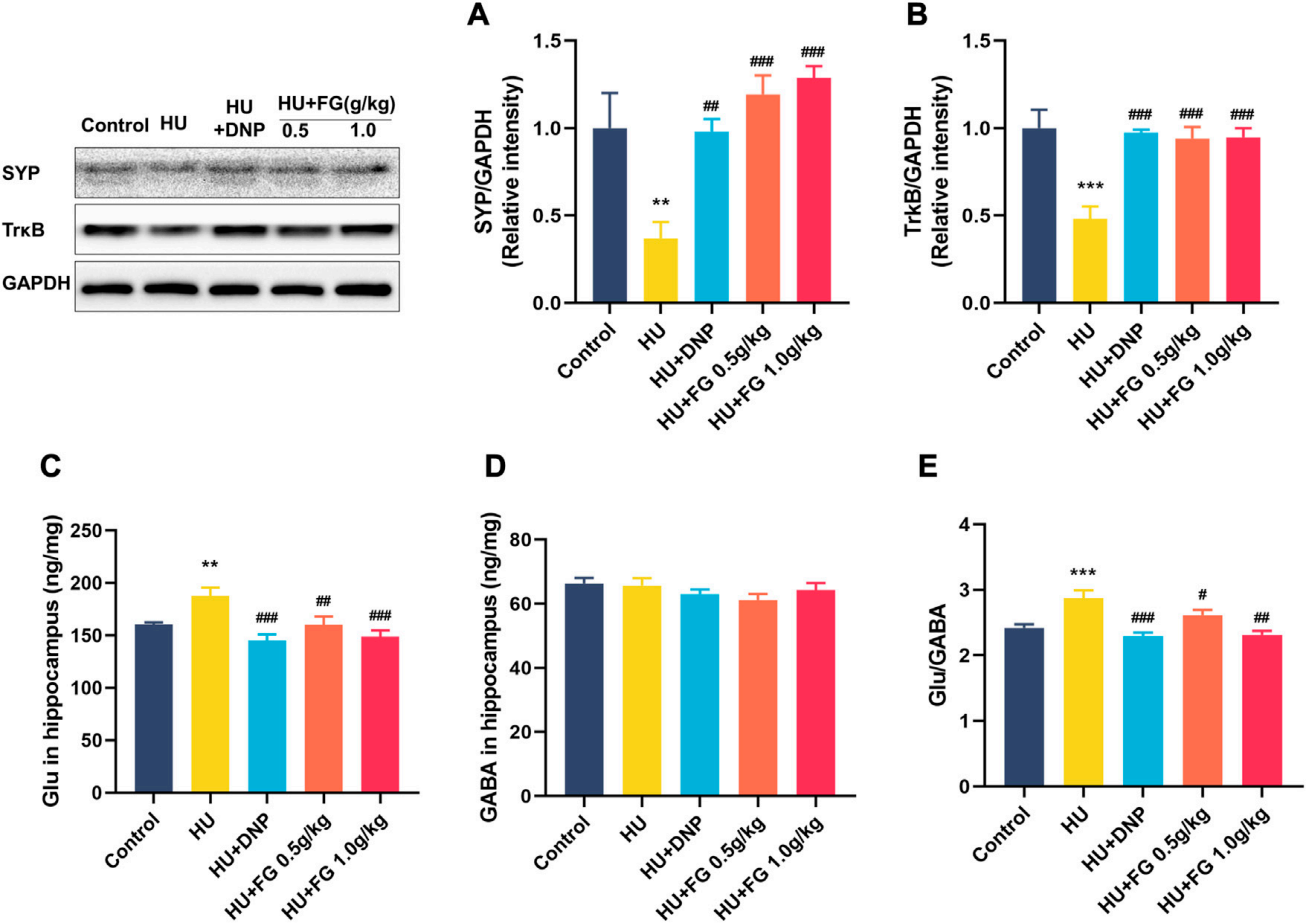
The effect of FG on the anti-apoptotic effect and maintaining the stability of neurotransmitters in the hippocampus. **(A)** The protein expression of SYP and **(B)**TrkB (n = 3). **(C)** The levels of Glu and **(D)** GABA. **(E)** The ratio of Glu/GABA (n = 8–9). Data were expressed as mean ± SEM. **p < 0.01 and ***p < 0.001 versus the control group; ^#^ *p <* 0.05, ^##^ *p <* 0.01, and ^###^ *p <* 0.001 versus the HU group.

Figure 7 showed that the expression of SYP and TrkB in the HU group was significantly reduced (*p <* 0.01 and *p <* 0.001), while the DNP and FG (both low and high dose) administration could significantly reverse these reductions (*p <* 0.01 and *p <* 0.001). The level of Glu in the HU group was significantly increased (F(4,35) = 6.603, *p <* 0.01) and affected the balance of GABA/GLU (F(4,35) = 9.749, *p <* 0.001). After administration of DNP and FG (both low and high doses), the abnormal increase of Glu and the GABA/Glu ratio was reversed (*p <* 0.001 and *p <* 0.05).

## Discussion

In the present study, we identified the cognitive improvement effect of the fresh *Gastrodia elata* Blume (FG), which alleviated hindlimb unloading (HU) and induced spatial and working cognitive dysfunction in mice. Specifically, FG improved the performance of mice in the object location recognition test (OLRT), Step-Down test (SDT), and Morris Water Maze test (MWMT). In biochemical experiments, we found that being given FG, the oxidative stress in the serum and neuroinflammatory response in the hippocampus induced by HU were suppressed. Meanwhile, the hippocampal apoptosis-related proteins decreased with the administration of FG, which may be mediated by the upregulation of the PI3K/AKT signaling pathway. In addition, it is also found that FG played a role in improving synaptic plasticity and neurotransmitter transmission.

*Gastrodia elata* Blume belongs to the genus Gastrodia in the orchid family. In modern pharmacological research, it is known as a popular traditional medicine for the treatment of neurological diseases including headache, dizziness, Alzheimer’s disease and Parkinson’s disease, and so on, and for its distinguished neuroprotective effect (Lu et al., 2022). The application of fresh *Gastrodia elata* was first seen in the *Shennong Herbal Classic*, which is also a common edible or medicinal form for the public (Huang et al., 2022); whereas, the main research form of *Gastrodia elata* Blume is generally processed products, and the application of fresh *Gastrodia elata* Blume (FG) is less. In this research, FG is obtained from the expressed juice of fresh *Gastrodia elata* Blume and is filtered, freeze-dried, and stored as powder until used. Our previous studies have proved the remarkable effect of FG in improving cognitive impairment caused by chronic stress, including circadian rhythm disorder and restraint (Huang et al., 2021; Huang et al., 2022). Together with weightlessness, all these are characteristic of the extreme environments that astronauts are faced with during spaceflight. Therefore, it is of great meaning to study the protective effect of FG on cognitive impairment caused by stimulated weightlessness, which not only provides scientific evidence for FG to ameliorate cognitive dysfunction in special space environment but also enriches the application of *Gastrodia elata* Blum as a health product.

HU is a classic animal model simulating microgravity on Earth to study aerospace medicine. The present work shows that after 28 days of HU modeling, the short-term and long-term memory of animals is weakened. OLRT has been used extensively to detect short-term spatial memory (Arbogast et al., 2019). The perception ability of mice to the new place of the object declined, as evidenced by decreased discrimination index. In SDT, animals would actively escape to the insulated platform to avoid injuries after learning that electrical stimulation continuously exists on the ground (Hiramatsu et al., 1995). As evidenced by the decline in escape latency and error times, the HU group showed a weakened short-term spatial memory in passive conditions. In MWMT, a classic spatial learning and memory testing method, animals can only rely on spatial reference to find the location of the platform which is hidden in a fixed position under the water surface (Morris, 1984). In the positioning navigation training, mice in the HU group escaping from the aversive water displayed increased escape latency, swimming distance, and the ratio of both time and distance spent in the target quadrant, indicating that an impairment occurred to the mice in their learning and memory abilities. At the stage of the space exploration experiment, the platform originally fixed in the target quadrant was removed, and the trained animals were expected to pass through the positions where the platform originally existed. Similarly, the cognitive impairment in the HU group was verified by the reduction of crossing times. In the working memory experiment, the platform originally fixed in the first quadrant was moved into the adjacent quadrant to detect the working memory of mice, and the prolonged escape latency showed that the working ability of mice was damaged by HU modeling. The above results suggested that HU could cause cognitive impairment in mice, which were consistent with the results reported in previous studies (Sun et al., 2009; Zhang et al., 2018; Li et al., 2021). The behavior deficits were corrected by the administration of FG (both 0.5 g/kg and 1.0 kg/kg), which indicated FG’s beneficial effects on HU-induced cognitive impairment of mice.

Oxidative stress injury occurs when people suffered from exogenous acute or chronic stress, leading to cell dysfunction and apoptosis (Rohleder, 2019; Forman and Zhang, 2021; Xue et al., 2022). The level of oxidative stress-related markers in the serum is found to be altered in the serum of patients with memory impairment (Du et al., 2019; Torres et al., 2021). Studies have proved that the indicators related to oxidative stress were changed in the serum of astronauts including the peroxide oxidation of lipids (POL), oxidized low-density lipoprotein (ox-LDL), and so on (Markin et al., 1997; Lee et al., 2020). In rodent experiments in space and on earth, antioxidant defense genes such as Ehd2 and oxidative stress-related biomarkers such as superoxide dismutase (SOD) and malondialdehyde (MDA) changed significantly (Mao et al., 2014; Moustafa, 2021). SOD is the most important enzyme that protects against the damage of reactive oxygen species or free radicals in organisms (Ma et al., 2021). MDA is one of the main products of cell membrane oxidation, which is used as a biomarker of peroxidation (An et al., 2022). The present study found that the HU procedure could reduce the level of antioxidant substances SOD while increasing the level of oxidant substances including MDA and glutathione disulfide (GSSG) in mice serum. GSSG, the oxidized form of glutathione, is a tripeptide thiol antioxidant and plays an important role in cell oxidation and signal transduction (Rahman et al., 2006). The results are consistent with the previous research results, suggesting the reliability of HU modeling, while FG administration reversed these oxidative stress products and thus played an antioxidant role.

Neuroinflammation is closely associated with cognitive impairment in many pathological conditions. Studies have shown that stimulated weightlessness caused neuroinflammation in the brain and led to spatial memory disorder in mice (Qiong et al., 2016). When the body gets injured, microglia rapidly proliferate and activate, releasing a variety of pro-inflammatory factors including TNF- α, IL-6, and INF- γ, etc. (Muhammad et al., 2019), while anti-inflammatory factors such as Arg-1, IL-10, and IL-4 can antagonize inflammatory reactions and inhibit astrocyte activation (Zhang and Wei, 2020; Descalzi, 2021). Astrocytes can release nuclear factor-κB (NF-κB) triggered by inflammatory mediators, which is known as an important transcription factor in inflammation, releasing various pro-inflammatory factors and leading to neuroinflammation in its over-activation situation (Dresselhaus and Meffert, 2019; Gao et al., 2021; Peng et al., 2021). According to our result, FG therapy had the function of maintaining the balance of pro-inflammation and anti-inflammation in the hippocampus by regulating the abnormal changes of the above oxidative stress-related factors. In addition, the expressions of NF-κB and NOD-like receptor protein 3 (NLRP3), both of which are essential drivers of inflammation, were inhibited by FG administration. NLRP3 inflammasome belongs to the NLR family and is the representative component of the innate immune system, which can be activated by NF-κB. Being over-activated, NLRP3 can release inflammatory factors and mediate downstream inflammatory reactions (Jiang et al., 2022). Many studies have shown that the activation of NLRP3 has a close association with cognitive dysfunction that can be attenuated by inhibiting NLRP3 (Ye et al., 2018; Garaschuk, 2021). Inhibiting the NLRP3/NF-κB pathway plays an important role in blocking the occurrence of neuroinflammation caused by stress injury. Therefore, we suggest that FG may play an anti-inflammatory role by inhibiting the activation of NLRP3/NF-κB pathway, thereby reducing HU-induced cognitive impairment.

A notable increase in the level of apoptosis-related proteins in the hippocampus was caused by the hindlimb unloading procedure in the present study. Previous research has proved that apoptosis in the brain occurs after long-term simulated weightlessness and is accompanied by mitochondrial metabolic abnormalities (Nguyen et al., 2021). During cellular stress, the pro-apoptotic protein Bax transfers from the cytoplasm to the membrane of the mitochondria through translocation, thus improving the permeability of the outer membrane of the mitochondria (Spitz et al., 2021). As induced by Bax, Cytochrome C (Cyt C) gets released into the cytoplasm, activating the apoptosis-related cascade reaction and finally leading to programmed cell death (Kulikov et al., 2012). Mitochondria are the main place for providing energy for cells. The abnormal elevation of dynamin-related protein 1 (Drp1), which is an important protein to maintain the balance of mitochondrial fusion and division, was found in neurological diseases, leading to neuronal injury (Feng et al., 2020). Besides, it was found that preventing mitochondrial dysfunction by inhibiting Drp1 could protect neurons from damage caused by oxidative stress (Oliver and Reddy, 2019). This study found that FG can downregulate the levels of Cyt C, Bax, and Drp1 in the hippocampus increased by HU modeling, thus, inhibiting the apoptosis of nerve cells. Moreover, the results showed that HU caused an increase in the levels of phosphatidylinositol3-kinase (PI3K), protein kinase B (AKT), and mammalian target of rapamycin (mTOR) in the hippocampus, and according to the Nissl staining’s results, FG therapy improved the arrangement and loss of neurons. PI3K/AKT as a classic signaling pathway plays an essential role in affecting various biological activities including the regulation of cell survival, metabolism and apoptosis, and so on due to its downstream protein participating in the transcription of a large number of genes and protein expression (Ediriweera et al., 2019). When the body receives external stimulation, PI3K phosphorylation is activated and acts on downstream targets, of which AKT is the prominent downstream effector. The activation of this PI3K/AKT pathway brings the increase of apoptosis-related proteins such as Bcl-2, Bax, etc., thus, causing programmed cell death (Wang et al., 2020). Its downstream signal molecule mTOR is considered an important protein in regulating the survival, differentiation, and maturation of neurons, and the upregulation of mTOR is proven to be beneficial to AD pathologies (Van Skike et al., 2020; Shi et al., 2022). In addition, cell apoptosis can be induced via inhibiting the PI3K/Akt/mTOR pathway (Yang et al., 2018; Chang X. et al., 2021). Taken together, our results demonstrated that the cognitive improvement effect of FG may be achieved through the activation of the PI3K/AKT/mTOR pathway, thus, inhibiting the release of apoptotic proteins and ultimately protecting nerve cells.

An increasing number of studies have proved the connection between synaptic plasticity and cognition (Raven et al., 2018; Santello et al., 2019). Long-term space microgravity environment can affect the main mediators related to brain plasticities such as 5-HT and BDNF (Popova et al., 2020). Synaptophysin (SYP) as a major membrane protein regulates the endocytosis of synaptic vesicles and has an obvious influence on the change of synaptic transmission efficiency (Chang C.-W. et al., 2021). Research has found that SYP decreased and pro-inflammatory factors increased in mice with Alzheimer’s disease, and increasing the expression of SYP can ameliorate the impairment of the synaptic transmission effect in cognitive impairment (Liu et al., 2020; Jiang et al., 2021). Tyrosine Kinase receptor B (TrkB) is located in the postsynaptic membrane and is a functional receptor of brain-derived neurotrophic factor (BDNF) (Chen et al., 2021). BDNF needs to be combined with TrkB to activate the intracellular signal transduction pathway, thus, producing corresponding molecules to protect neurons and promote regeneration (Mitre et al., 2022). The present results showed that FG therapy significantly increased the expression of SYP and TrkB. In addition, the abnormal increase of Glu in the hippocampus was suppressed by FG, thus, maintaining the balance of glutamate/GABA. Glutamate and GABA are the main excitatory and inhibitory neurotransmitters, respectively, in the brain, and maintaining their balance assists in the smooth operation of the nervous system (Sood et al., 2021). Previous studies have proved that the abnormal increase of glutamate and the imbalance of glutamate/GABA occurred in the hippocampus of chronic simulated microgravity rats, and this procedure was mediated by presynaptic proteins, which is consistent with our results (Wang et al., 2015). Therefore, we suggest that FG has beneficial effects on improving synaptic plasticity and alleviating the excitatory toxicity of glutamate.

## Conclusion

The present study provides evidence for the first time that FG can effectively improve HU-induced cognitive impairment in mice. The cognitive-enhancing effect of FG may be related to the inhibition of NF-κB and NLRP3 to reduce neuroinflammation and the improvement of the PI3K/Akt/mTOR pathway to alleviate neuronal apoptosis. The above results indicate that FG, as a health food, has great therapeutic potential in protecting against cognitive impairment caused by special space environments.

## Data Availability

The raw data supporting the conclusion of this article will be made available by the authors, without undue reservation.

## References

[B1] An J.-R. Su J.-N. Sun G.-Y. Wang Q.-F. Fan Y.-D. Jiang N. (2022). Liraglutide alleviates cognitive deficit in db/db mice: Involvement in oxidative stress, iron overload, and ferroptosis. Neurochem. Res. 47 (2), 279–294. 10.1007/s11064-021-03442-7 34480710

[B2] Arbogast T. Razaz P. Ellegood J. McKinstry S. U. Erdin S. Currall B. (2019). Kctd13-deficient mice display short-term memory impairment and sex-dependent genetic interactions. Hum. Mol. Genet. 28 (9), 1474–1486. 10.1093/hmg/ddy436 30590535 PMC6489413

[B3] Bock O. Weigelt C. Bloomberg J. J. (2010). Cognitive demand of human sensorimotor performance during an extended space mission: A dual-task study. Aviat. Space, Environ. Med. 81 (9), 819–824. 10.3357/asem.2608.2010 20824987

[B4] Casler J. G. Cook J. R. (1999). Cognitive performance in space and analogous environments. Int. J. Cognitive Ergonomics 3 (4), 351–372. 10.1207/s15327566ijce0304_5 11543512

[B5] Chang C.-W. Hsiao Y.-T. Jackson M. B. (2021). Synaptophysin regulates fusion pores and exocytosis mode in chromaffin cells. J. Neurosci. Official J. Soc. For Neurosci. 41 (16), 3563–3578. 10.1523/JNEUROSCI.2833-20.2021 PMC8055083 33664131

[B6] Chang X. Wang X. Li J. Shang M. Niu S. Zhang W. (2021). Silver nanoparticles induced cytotoxicity in HT22 cells through autophagy and apoptosis via PI3K/AKT/mTOR signaling pathway. Ecotoxicol. Environ. Saf. 208, 111696. 10.1016/j.ecoenv.2020.111696 33396027

[B7] Chen C. Ahn E. H. Liu X. Wang Z.-H. Luo S. Liao J. (2021). Optimized TrkB agonist ameliorates Alzheimer's disease pathologies and improves cognitive functions via inhibiting delta-secretase. ACS Chem. Neurosci. 12 (13), 2448–2461. 10.1021/acschemneuro.1c00181 34106682 PMC8269693

[B8] Descalzi G. (2021). Cortical astrocyte-neuronal metabolic coupling emerges as a critical modulator of stress-induced hopelessness. Neurosci. Bull. 37 (1), 132–134. 10.1007/s12264-020-00559-7 32789727 PMC7811966

[B9] Dresselhaus E. C. Meffert M. K. (2019). Cellular specificity of NF-κB function in the nervous system. Front. Immunol. 10, 1043. 10.3389/fimmu.2019.01043 31143184 PMC6520659

[B10] Du L. Ma J. He D. Zhang X. (2019). Serum ischaemia-modified albumin might be a potential biomarker for oxidative stress in amnestic mild cognitive impairment. Psychogeriatrics Official J. Jpn. Psychogeriatr. Soc. 19 (2), 150–156. 10.1111/psyg.12377 30362220

[B11] Ediriweera M. K. Tennekoon K. H. Samarakoon S. R. (2019). Role of the PI3K/AKT/mTOR signaling pathway in ovarian cancer: Biological and therapeutic significance. Seminars Cancer Biol. 59, 147–160. 10.1016/j.semcancer.2019.05.012 31128298

[B12] Fasina O. B. Wang J. Mo J. Osada H. Ohno H. Pan W. (2022). Gastrodin from gastrodia elata enhances cognitive function and neuroprotection of AD mice via the regulation of gut microbiota composition and inhibition of neuron inflammation. Front. Pharmacol. 13, 814271. 10.3389/fphar.2022.814271 35721206 PMC9201506

[B13] Feng S.-T. Wang Z.-Z. Yuan Y.-H. Wang X.-L. Sun H.-M. Chen N.-H. (2020). Dynamin-related protein 1: A protein critical for mitochondrial fission, mitophagy, and neuronal death in Parkinson's disease. Pharmacol. Res. 151, 104553. 10.1016/j.phrs.2019.104553 31760107

[B14] Forman H. J. Zhang H. (2021). Targeting oxidative stress in disease: Promise and limitations of antioxidant therapy. Nat. Rev. Drug Discov. 20 (9), 689–709. 10.1038/s41573-021-00233-1 34194012 PMC8243062

[B15] Fowler B. Comfort D. Bock O. (2000). A review of cognitive and perceptual-motor performance in space. Aviat. Space, Environ. Med. 71 (9), A66–A68. 10993312

[B16] Gao W. Ning Y. Peng Y. Tang X. Zhong S. Zeng H. (2021). LncRNA NKILA relieves astrocyte inflammation and neuronal oxidative stress after cerebral ischemia/reperfusion by inhibiting the NF-κB pathway. Mol. Immunol. 139, 32–41. 10.1016/j.molimm.2021.08.002 34454183

[B17] Garaschuk O. (2021). The role of NLRP3 inflammasome for microglial response to peripheral inflammation. Neural Regen. Res. 16 (2), 294–295. 10.4103/1673-5374.290889 32859781 PMC7896234

[B18] Globus R. K. Morey-Holton E. (2016). Hindlimb unloading: Rodent analog for microgravity. J. Appl. Physiology 120 (10), 1196–1206. 10.1152/japplphysiol.00997.2015 26869711

[B19] Hiramatsu M. Sasaki M. Kameyama T. (1995). Effects of dynorphin A-(1-13) on carbon monoxide-induced delayed amnesia in mice studied in a step-down type passive avoidance task. Eur. J. Pharmacol. 282 (1-3), 185–191. 10.1016/0014-2999(95)00330-n 7498274

[B20] Homick J. L. Delaney P. Rodda K. (1998). Overview of the neurolab spacelab mission. Acta Astronaut. 42 (1-8), 69–87. 10.1016/s0094-5765(98)00107-6 11541633

[B21] Huang H. Jiang N. Zhang Y. W. Lv J. W. Wang H. X. Lu C. (2021). Gastrodia elata blume ameliorates circadian rhythm disorder-induced mice memory impairment. Life Sci. Space Res. (Amst) 31, 51–58. 10.1016/j.lssr.2021.07.004 34689950

[B22] Huang H. Zhang Y. Yao C. He Q. Chen F. Yu H. (2022). The effects of fresh Gastrodia elata Blume on the cognitive deficits induced by chronic restraint stress. Front. Pharmacol. 13, 890330. 10.3389/fphar.2022.890330 36105220 PMC9464977

[B23] Jiang N. Zhang Y. Yao C. Huang H. Wang Q. Huang S. (2022). Ginsenosides Rb1 attenuates chronic social defeat stress-induced depressive behavior via regulation of SIRT1-NLRP3/nrf2 pathways. Front. Nutr. 9, 868833. 10.3389/fnut.2022.868833 35634375 PMC9133844

[B24] Jiang Y. Li K. Li X. Xu L. Yang Z. (2021). Sodium butyrate ameliorates the impairment of synaptic plasticity by inhibiting the neuroinflammation in 5XFAD mice. Chemico-biological Interact. 341, 109452. 10.1016/j.cbi.2021.109452 33785315

[B25] Kulikov A. V. Shilov E. S. Mufazalov I. A. Gogvadze V. Nedospasov S. A. Zhivotovsky B. (2012). Cytochrome c: The achilles' heel in apoptosis. Cell. Mol. Life Sci. CMLS 69 (11), 1787–1797. 10.1007/s00018-011-0895-z 22179840 PMC11114681

[B26] Lee S. M. C. Ribeiro L. C. Martin D. S. Zwart S. R. Feiveson A. H. Laurie S. S. (2020). Arterial structure and function during and after long-duration spaceflight. J. Appl. Physiology 129 (1), 108–123. 10.1152/japplphysiol.00550.2019 32525433

[B27] Li Q. Yan J. Liao J. Zhang X. Liu L. Fu X. (2021). Distinct effects of social stress on working memory in obsessive-compulsive disorder. Neurosci. Bull. 37 (1), 81–93. 10.1007/s12264-020-00579-3 33000423 PMC7811969

[B28] Lin Y. E. Lin C. H. Ho E. P. Ke Y. C. Petridi S. Elliott C. J. (2021). Glial Nrf2 signaling mediates the neuroprotection exerted by Gastrodia elata Blume in Lrrk2-G2019S Parkinson's disease. Elife 10, e73753. 10.7554/eLife.73753 34779396 PMC8660019

[B29] Liu B. Kou J. Li F. Huo D. Xu J. Zhou X. (2020). Lemon essential oil ameliorates age-associated cognitive dysfunction via modulating hippocampal synaptic density and inhibiting acetylcholinesterase. Aging 12 (9), 8622–8639. 10.18632/aging.103179 32392535 PMC7244039

[B30] Lu C. Qu S. Zhong Z. Luo H. Lei S. S. Zhong H.-J. (2022). The effects of bioactive components from the rhizome of gastrodia elata blume (Tianma) on the characteristics of Parkinson's disease. Front. Pharmacol. 13, 963327. 10.3389/fphar.2022.963327 36532787 PMC9748092

[B31] Lv J. Jiang N. Wang H. Huang H. Bao Y. Chen Y. (2021). Simulated weightlessness induces cognitive changes in rats illustrated by performance in operant conditioning tasks. Life Sci. Space Res. 29, 63–71. 10.1016/j.lssr.2021.03.004 33888289

[B32] Ma X. Song M. Yan Y. Ren G. Hou J. Qin G. (2021). Albiflorin alleviates cognitive dysfunction in STZ-induced rats. Aging 13 (14), 18287–18297. 10.18632/aging.203274 34319254 PMC8351685

[B33] Mao X. N. Zhou H. J. Yang X. J. Zhao L. X. Kuang X. Chen C. (2017). Neuroprotective effect of a novel gastrodin derivative against ischemic brain injury: Involvement of peroxiredoxin and TLR4 signaling inhibition. Oncotarget 8 (53), 90979–90995. 10.18632/oncotarget.18773 29207618 PMC5710899

[B34] Mao X. W. Pecaut M. J. Stodieck L. S. Ferguson V. L. Bateman T. A. Bouxsein M. L. (2014). Biological and metabolic response in STS-135 space-flown mouse skin. Free Radic. Res. 48 (8), 890–897. 10.3109/10715762.2014.920086 24796731

[B35] Markin A. A. Popova I. A. Vetrova E. G. Zhuravleva O. A. Balashov O. I. (1997). Lipid peroxidation and activity of diagnostically significant enzymes in cosmonauts after flights of various durations. Aviakosmicheskaia I Ekologicheskaia Meditsina = Aerosp. Environ. Med. 31 (3), 14–18. 9244500

[B36] Mitre M. Saadipour K. Williams K. Khatri L. Froemke R. C. Chao M. V. (2022). Transactivation of TrkB receptors by oxytocin and its G protein-coupled receptor. Front. Mol. Neurosci. 15, 891537. 10.3389/fnmol.2022.891537 35721318 PMC9201241

[B37] Morris R. (1984). Developments of a water-maze procedure for studying spatial learning in the rat. J. Neurosci. Methods 11 (1), 47–60. 10.1016/0165-0270(84)90007-4 6471907

[B38] Moustafa A. (2021). Hindlimb unloading-induced reproductive suppression via Downregulation of hypothalamic Kiss-1 expression in adult male rats. Reproductive Biol. Endocrinol. RB&E 19 (1), 37. 10.1186/s12958-021-00694-4 33663539 PMC7931529

[B39] Muhammad T. Ikram M. Ullah R. Rehman S. U. Kim M. O. (2019). Hesperetin, a citrus flavonoid, attenuates LPS-induced neuroinflammation, apoptosis and memory impairments by modulating TLR4/NF-κB signaling. Nutrients 11 (3), 648. 10.3390/nu11030648 30884890 PMC6471991

[B40] Ng C. F. Ko C. H. Koon C. M. Chin W. C. Kwong H. C. Lo A. W. (2016). The aqueous extract of rhizome of Gastrodia elata Blume attenuates locomotor defect and inflammation after traumatic brain injury in rats. J. Ethnopharmacol. 185, 87–95. 10.1016/j.jep.2016.03.018 26979339

[B41] Nguyen H. P. Tran P. H. Kim K.-S. Yang S.-G. (2021). The effects of real and simulated microgravity on cellular mitochondrial function. NPJ Microgravity 7 (1), 44. 10.1038/s41526-021-00171-7 34750383 PMC8575887

[B42] Oliver D. Reddy P. H. (2019). Dynamics of dynamin-related protein 1 in Alzheimer's disease and other neurodegenerative diseases. Cells 8 (9), 961. 10.3390/cells8090961 31450774 PMC6769467

[B43] Peng Z. Li X. Li J. Dong Y. Gao Y. Liao Y. (2021). Dlg1 knockout inhibits microglial activation and alleviates lipopolysaccharide-induced depression-like behavior in mice. Neurosci. Bull. 37 (12), 1671–1682. 10.1007/s12264-021-00765-x 34490521 PMC8643377

[B44] Popova N. K. Kulikov A. V. Naumenko V. S. (2020). Spaceflight and brain plasticity: Spaceflight effects on regional expression of neurotransmitter systems and neurotrophic factors encoding genes. Neurosci. Biobehav. Rev. 119, 396–405. 10.1016/j.neubiorev.2020.10.010 33086127

[B45] Qiong W. Yong-Liang Z. Ying-Hui L. Shan-Guang C. Jiang-Hui G. Yi-Xi C. (2016). The memory enhancement effect of Kai Xin San on cognitive deficit induced by simulated weightlessness in rats. J. Ethnopharmacol. 187, 9–16. 10.1016/j.jep.2016.03.070 27103112

[B46] Rahman I. Kode A. Biswas S. K. (2006). Assay for quantitative determination of glutathione and glutathione disulfide levels using enzymatic recycling method. Nat. Protoc. 1 (6), 3159–3165. 10.1038/nprot.2006.378 17406579

[B47] Raven F. Van der Zee E. A. Meerlo P. Havekes R. (2018). The role of sleep in regulating structural plasticity and synaptic strength: Implications for memory and cognitive function. Sleep. Med. Rev. 39, 3–11. 10.1016/j.smrv.2017.05.002 28641933

[B48] Rohleder N. (2019). Stress and inflammation - the need to address the gap in the transition between acute and chronic stress effects. Psychoneuroendocrinology 105, 164–171. 10.1016/j.psyneuen.2019.02.021 30826163

[B49] Santello M. Toni N. Volterra A. (2019). Astrocyte function from information processing to cognition and cognitive impairment. Nat. Neurosci. 22 (2), 154–166. 10.1038/s41593-018-0325-8 30664773

[B50] Shi Q. Chang C. Saliba A. Bhat M. A. (2022). Microglial mTOR activation upregulates Trem2 and enhances β-amyloid plaque clearance in the 5XFAD Alzheimer's disease model. J. Neurosci. Official J. Soc. For Neurosci. 42 (27), 5294–5313. 10.1523/JNEUROSCI.2427-21.2022 PMC9270922 35672148

[B51] Sood A. Preeti K. Fernandes V. Khatri D. K. Singh S. B. (2021). Glia: A major player in glutamate-GABA dysregulation-mediated neurodegeneration. J. Neurosci. Res. 99 (12), 3148–3189. 10.1002/jnr.24977 34748682

[B52] Spitz A. Z. Zacharioudakis E. Reyna D. E. Garner T. P. Gavathiotis E. (2021). Eltrombopag directly inhibits BAX and prevents cell death. Nat. Commun. 12 (1), 1134. 10.1038/s41467-021-21224-1 33602934 PMC7892824

[B53] Sun X.-Q. Xu Z.-P. Zhang S. Cao X.-S. Liu T.-S. (2009). Simulated weightlessness aggravates hypergravity-induced impairment of learning and memory and neuronal apoptosis in rats. Behav. Brain Res. 199 (2), 197–202. 10.1016/j.bbr.2008.11.035 19100783

[B54] Torres M. L. Wanionok N. E. McCarthy A. D. Morel G. R. Fernández J. M. (2021). Systemic oxidative stress in old rats is associated with both osteoporosis and cognitive impairment. Exp. Gerontol. 156, 111596. 10.1016/j.exger.2021.111596 34678425

[B55] Van Skike C. E. Lin A.-L. Roberts Burbank R. Halloran J. J. Hernandez S. F. Cuvillier J. (2020). mTOR drives cerebrovascular, synaptic, and cognitive dysfunction in normative aging. Aging Cell 19 (1), e13057. 10.1111/acel.13057 31693798 PMC6974719

[B56] Wang L.-S. Zhang M.-D. Tao X. Zhou Y.-F. Liu X.-M. Pan R.-L. (2019). LC-MS/MS-based quantification of tryptophan metabolites and neurotransmitters in the serum and brain of mice. J. Chromatogr. B, Anal. Technol. Biomed. Life Sci. 1112, 24–32. 10.1016/j.jchromb.2019.02.021 30836315

[B57] Wang M.-Y. Meng M. Yang C.-C. Zhang L. Li Y.-L. Zhang L. (2020). Cornel iridoid glycoside improves cognitive impairment induced by chronic cerebral hypoperfusion via activating PI3K/Akt/GSK-3β/CREB pathway in rats. Behav. Brain Res. 379, 112319. 10.1016/j.bbr.2019.112319 31669346

[B58] Wang Y. Iqbal J. Liu Y. Su R. Lu S. Peng G. (2015). Effects of simulated microgravity on the expression of presynaptic proteins distorting the GABA/glutamate equilibrium--A proteomics approach. Proteomics 15 (22), 3883–3891. 10.1002/pmic.201500302 26359799

[B59] Xue X.-J. Su R. Li Z.-F. Bu X.-O. Dang P. Yu S.-F. (2022). Oxygen metabolism-induced stress response underlies heart-brain interaction governing human consciousness-breaking and attention. Neurosci. Bull. 38 (2), 166–180. 10.1007/s12264-021-00761-1 34435318 PMC8821743

[B60] Yang J. Pi C. Wang G. (2018). Inhibition of PI3K/Akt/mTOR pathway by apigenin induces apoptosis and autophagy in hepatocellular carcinoma cells. Biomed. Pharmacother. 103, 699–707. 10.1016/j.biopha.2018.04.072 29680738

[B61] Yang Y. Li S. Huang H. Lv J. Chen S. Pires Dias A. C. (2020). Comparison of the protective effects of ginsenosides Rb1 and Rg1 on improving cognitive deficits in SAMP8 mice based on anti-neuroinflammation mechanism. Front. Pharmacol. 11, 834. 10.3389/fphar.2020.00834 32587516 PMC7298198

[B62] Ye T. Meng X. Wang R. Zhang C. He S. Sun G. (2018). Gastrodin alleviates cognitive dysfunction and depressive-like behaviors by inhibiting ER stress and NLRP3 inflammasome activation in db/db mice. Int. J. Mol. Sci. 19 (12), 3977. 10.3390/ijms19123977 30544722 PMC6321309

[B63] Zhang L. Wei W. (2020). Anti-inflammatory and immunoregulatory effects of paeoniflorin and total glucosides of paeony. Pharmacol. Ther. 207, 107452. 10.1016/j.pharmthera.2019.107452 31836457

[B64] Zhang Y. Wang Q. Chen H. Liu X. Lv K. Wang T. (2018). Involvement of cholinergic dysfunction and oxidative damage in the effects of simulated weightlessness on learning and memory in rats. BioMed Res. Int. 2018, 2547532. 10.1155/2018/2547532 29581965 PMC5822892

[B65] Zhou B. Tan J. Zhang C. Wu Y. (2018). Neuroprotective effect of polysaccharides from Gastrodia elata blume against corticosterone-induced apoptosis in PC12 cells via inhibition of the endoplasmic reticulum stress-mediated pathway. Mol. Med. Rep. 17 (1), 1182–1190. 10.3892/mmr.2017.7948 29115511

